# The Irr1/Scc3 protein implicated in chromosome segregation in *Saccharomyces cerevisiae* has a dual nuclear-cytoplasmic localization

**DOI:** 10.1186/s13008-016-0027-0

**Published:** 2017-01-03

**Authors:** Piotr Kowalec, Jan Fronk, Anna Kurlandzka

**Affiliations:** 1 Institute of Biochemistry and Biophysics, Polish Academy of Sciences, Pawinskiego 5A, 02–106 Warsaw, Poland; 2 Department of Molecular Biology, Faculty of Biology, Institute of Biochemistry, University of Warsaw, Miecznikowa 1, 02-096 Warsaw, Poland

**Keywords:** Chromosome segregation, Irr1/Scc3, Cohesin complex, *Saccharomyces cerevisiae*

## Abstract

**Background:**

Correct chromosome segregation depends on the sister chromatid cohesion complex. The essential, evolutionarily conserved regulatory protein Irr1/Scc3, is responsible for the complex loading onto DNA and for its removal. We found that, unexpectedly, Irr1 is present not only in the nucleus but also in the cytoplasm.

**Results:**

We show that Irr1 protein is enriched in the cytoplasm upon arrest of yeast cells in G1 phase following nitrogen starvation, diauxic shift or α-factor action, and also during normal cell cycle. Despite the presence of numerous Crm1-dependent export signals, the cytoplasmic pool of Irr1 is not derived through export from the nucleus but instead is simply retained in the cytoplasm. Cytoplasmic Irr1 interacts with the Imi1 protein implicated in glutathione homeostasis and mitochondrial integrity.

**Conclusions:**

Besides regulation of the sister chromatid cohesion complex in the nucleus Irr1 appears to have an additional role in the cytoplasm, possibly through interaction with the cytoplasmic protein Imi1.

## Background

Correct chromosome segregation during cell division is ensured by a highly regulated series of events. Cohesin, an evolutionarily conserved and essential three-subunit DNA-associated protein complex, is an important element which safeguards the accurate segregation, although its role is not limited to chromatid cohesion only, as was supposed initially (reviewed in [1, 2]). In *Saccharomyces cerevisiae* the cohesin subunits are called Smc1, Smc3 and Mcd1/Scc1.

The cohesin complex tethers sister chromatids together until mitosis onset [3, 4]. It associates with and dissociates from chromosomes and its dynamics depends mainly on the loading complex Scc2–Scc4 and the regulatory subunits Wapl and Pds5 [3, 5]. In budding yeast cohesin associates with chromatin during the G1/S phase, whereas in vertebrates the binding takes place during telophase of the preceding cell cycle [6]. Additionally, double-strand DNA breaks induce cohesin binding with chromatin in G2/M phase. In metazoa cohesin complexes are found on chromosomes from telophase until anaphase onset and they bind in two modes which differ in stability [7]. Cohesin is removed from the chromosomes in a stepwise process that starts in prophase and is completed in anaphase. It is regulated by cell cycle-regulated kinases and requires the Wapl protein and proteolysis of the Mcd1 subunit (reviewed in [8]).

In addition to their canonical role in sister chromatid cohesion, cohesins are required for higher-order chromatin organization and play important roles in DNA repair and replication, as well as in gene regulation [9, 10]. The complexes that mediate chromatid cohesion are more stably attached to chromosomes than those regulating transcription during interphase [7]. Some studies show that cohesin may also have a role in apoptosis [11–14].

When the cell undergoes differentiation, it exits the G1 phase of the cell cycle to enter a quiescent state referred to as G0. The fate of cohesins in the G0 phase, where the differentiated cell spends most of its life, remains largely unknown, although cohesin is still associated with chromatin in quiescent cells [15–17].

The essential protein Irr1/Scc3 of *S. cerevisiae* was initially assigned to the cohesin complex [18–20]. However, early results from our laboratory suggested that it may play a regulatory role [21]. We also showed Irr1 involvement in regulating expression of genes linked to cell wall functioning [22, 23]. A closer examination of SA2, a mammalian homolog of yeast Irr1, indicated that it may act as a transcriptional co-activator by interacting with transcription factors [24]. Recently it became clear that Irr1 and its homologue in S*chizosaccharomyces pombe* Psc3^Scc3^ are important regulatory proteins responsible for cohesin complex loading onto DNA [25, 26]. It has also been postulated that Irr1/Scc3 participates in releasing the cohesin complex from DNA [27]. Establishing the exact role and mode of functioning of Irr1 seems worthwhile since all eukaryotic genomes encode orthologues of this essential protein.

The tight control of the cell cycle involves, among other modes, modulation of the subcellular distribution of relevant proteins between the nucleus and the cytoplasm. We previously noticed that SA2 is capable of nucleocytoplasmic shuttling and can be exported from the nucleus through a Crm1-dependent export pathway when expressed in yeast [28, 29]. The factors that provoke such shuttling and its role remain unknown.

Here we show that Irr1 protein can also be found outside the nucleus in *S. cerevisiae*, but not due to Crm1-dependent export. The cytoplasmic-to-nuclear proportion of Irr1 is the highest when cells are blocked in G1 phase by nitrogen starvation. We also describe in more detail the interaction between Irr1 and the cytoplasmic protein Imi1, involved in glutathione homeostasis, which we reported recently [30]. This interaction could be linked to the role of Irr1 in the cytoplasm.

## Methods

### Nomenclature, strains, media, growth conditions

Standard genetic nomenclature is used to designate wild-type alleles (e.g., *IRR1*, *URA3*), mutant alleles (e.g., *ade2*-*1*), and disruptants or deletions (e.g., *irr1::kanMX6*). Protein denoting is as follows: Irr1 encoded by *IRR1* gene. *S. cerevisiae* strains used in this study were derivatives of W303 and are listed in Table 1. Strains 2281 and 2239, obtained from the laboratory of Dr. Dmitri Ivanov (Bioinformatics Institute A*STAR, Singapore), had the *IRR1* gene originally named *SCC3*. Yeast culture media were prepared as described [31]. YPD medium contained 1% Bacto-yeast extract, 2% Bacto-peptone and 2% (all w/v) glucose. SD contained 0.67% yeast nitrogen base without amino acids (Difco) and 2% glucose. For auxotrophic strains, the media contained appropriate supplements. SD-N contained 0.17% yeast nitrogen base without amino acids and ammonium sulphate (Conda #1553), and 2% glucose. Standard methods were used for genetic manipulation of yeast [31]. Plasmid propagation was performed in chemically competent *Escherichia coli* XL1-Blue MRF’ (Stratagene).

**Table 1:** *Saccharomyces cerevisiae* strains

Strain	Genotype	Source
*IRR1*	MAT **a** *ade2*—*1 his3*-*11,15 leu2*-*3112 trp1*-*1 ura3*-*1 can1*-*100*	Rothstein collection (Columbia University, New York, USA)
2281^a^	MAT **a**/*α* {*ade2*-*1 his3*-*11,15 leu2*-*3,112 trp1*-*1 ura3*-*1 can1*-*100 irr1::IRR1*-*GFP/kanMX4 spc42::SPC42*-*mCherry/natMX6*}	D. Ivanov, Inst. Mol. Cell Biology, Singapore [25]
2239^a^	MAT **a** *ade2*-*1 his3*-*11,15 leu2*-*3,112 trp1*-*1 ura3*-*1 can1*-*100 irr1::IRR1*-*GFP/kanMX4 spc42::SPC42*-*mCherry/natMX6*	D. Ivanov
PJ69-4α	MAT *α trp1*-*901 leu2*-*3,112 ura3*-*52 his3*-*200 gal4Δ gal80Δ LYS2::GAL1*-*HIS3 GAL2*-*ADE2 met2::GAL7*-*lacZ*	[33]
Irr1-GFP/Imi1-RFP	MAT **a** *ade2*-*1 his3*-*11,15 leu2*-*3,112 trp1*-*1 ura3*-*1 can1*-*100 irr1::IRR1*-*GFP/kanMX4* [pCM189-*IMI1*-*RFP, URA3,* CEN]	This study, derivative of 2239
Irr1-GFP/RFP	MAT **a** *ade2*-*1 his3*-*11,15 leu2*-*3,112 trp1*-*1 ura3*-*1 can1*-*100 irr1::IRR1*-*GFP/kanMX4* [pCM189-*RFP, URA3,* CEN]	This study, derivative of 2239
GFP/Imi1-RFP	MAT **a** *ade2*-*1 his3*-*11,15 leu2*-*3,112 trp1*-*1 ura3*-*1 can1*-*100 imi1::natMX6* [pUG34-*GFP, HIS3,* CEN] [pCM189-*IMI1*-*RFP, URA3,* CEN]	This study, derivative of 2239
MNY7	MAT **a** *trp1 his3 leu2 ura3 crm1::kanR* [pDC-*CRM1, LEU2*, CEN]	[34]
MNY8	MAT **a** *trp1 his3 leu2 ura3 crm1::kanR* [pDC-*crm1*-T539C, *LEU2*, CEN]	[34]
MNY7-IRR1-GFP	MAT **a** *trp1 his3 leu2 ura3 crm1::kanR IRR1::irr1*-*GFP/kanMX4 spc42::SPC42*-*mCherry/natMX6* [pDC-*CRM1, LEU2*, CEN]	This study, derivative of MNY7
MNY8-IRR1-GFP	MAT **a** *trp1 his3 leu2 ura3 crm1::kanR IRR1::irr1*-*GFP/kanMX4 spc42::SPC42*-*mCherry/natMX6* [pDC- *crm1*-T539C, *LEU2*, CEN]	This study, derivative of MNY8
*irr1*(V248E)-*GFP*	MAT **a** *ade2*-*1 his3*-*11,15 leu2*-*3,112 trp1*-*1 ura3*-*1 can1*-*100 irr1:: kanMX4* [pUG35-*irr1*(V248E)-*GFP, URA3,* CEN]	This study, derivative of *IRR1*
*irr1*(F986A)-*GFP*	MAT **a** *ade2*-*1 his3*-*11,15 leu2*-*3112 trp1*-*1 ura3*-*1 can1*-*100 irr1:: kanMX4* [pUG35-*irr1*(F986A)-*GFP, URA3,* CEN]	This study, derivative of *IRR1*
*Irr1*(V248E, F986A)-*GFP*	MAT **a** *ade2*-*1 his3*-*11,15 leu2*-*3112 trp1*-*1 ura3*-*1 can1*-*100 irr1:: kanMX4* [pUG35-*irr1*(V248E, F986A)-*GFP, URA3,* CEN]	This study, derivative of *IRR1*

^a^In these strains the *IRR1* gene was originally named *SCC3* by Dr. Ivanov

Yeast nitrogen starvation was carried out according to [32] with modifications. Cells were grown in YPD to mid-log phase, centrifuged at 1000×*g* for 5 min, rinsed once with nitrogen starvation medium (SD-N), transferred to SD-N and incubated for 22 h with shaking. For Leptomycin B (LMB) experiments the chimaeric gene *IRR1*-*GFP* was introduced into MNY7 and MNY8 strains by genetic crosses with a MATα strain derived from 2281 by sporulation and tetrad dissection. Strains were grown overnight, then cultures were divided and one portion was treated with LMB (LC Laboratories, Woburn, MA, USA, cat. No. L-6100) at 40 ng per ml of medium and the second constituted a control. 1 h after LMB treatment the cells were collected, fixed with 4% formaldehyde and subjected to fluorescence microscopy.

### Yeast two-hybrid screen

The Irr1 two-hybrid bait comprised Gal4-DNA-BD-Irr1Δ1-467 and was expressed from the pGBKT7 plasmid (Clontech). The bait contained 683 C-terminal amino acids of Irr1, starting from I468. Direct two-hybrid analysis was done according to the protocols described by [35]. Growth on media lacking histidine was tested in the presence of 10 mM 3-amino-1,2,4-triazole. The host strain for two-hybrid studies, PJ69-4α [33], was transformed using the genomic library described by [36] and prepared from Ym955 strain provided by Dr. Mark Johnston (University of Colorado, USA).

### Plasmids

Plasmids listed in Table 2 were constructed by standard methods. All PCR products were sequenced after cloning.

**Table 2:** Plasmids used in this study

Plasmid	Description	Source:
pACTII	P_*ADH1*_-Gal4-AD-MCS- T_*ADH1*_ *Amp* ^*R*^ *LEU2* 2μ	Clontech #638822
pGBKT7	P_*ADH1*_-Gal4-DNA-BD-MCS-T_*T7 & ADH1*_ *Kan* ^*R*^ *TRP1* 2 μ	Clontech #630489
pGBKT7-C-Irr1	P_*ADH1*_-Gal4-DNA-BD-Irr1Δ1-474-T_*T7 & ADH1*_ *Kan* ^*R*^ *TRP1* 2μ	This study
pCM189	P_*tetO*_-MCS-T_*CYC1*_ *Amp* ^*R*^ *URA3* CEN	[37]
pCM189-P_*tetO*_-*IMI1*-*RFP*	P_*tetO*_-*IMI1*-*RFP*-T_*CYC1*_ *Amp* ^*R*^ *URA3* CEN	[30]
pCM189-P_*tetO*_-*RFP*	P_*tetO*_-*RFP*-T_*CYC1*_ *Amp* ^*R*^ *URA3* CEN	This study
pUG23	P_*MET25*_-MCS-*GFP*-T_*CYC1*_ *Amp* ^*R*^ *HIS3* CEN	U. Güldener and J. H. Hegemann, Heinrich-Heine-Universität, Düsseldorf, Germany
pUG34	P_*MET25*_-*GFP*-MCS-T_*CYC1*_ *Amp* ^*R*^ *HIS3* CEN	

### Whole cell lysates, cell fractionation and Western blot analyses

For whole-cell lysates yeast were grown in liquid medium and an equivalent of 5 OD_600_ units was harvested by centrifugation. Samples of whole-cell proteins were prepared by extraction of proteins achieved by 10-min incubation in 1.85 M NaOH and 7.4% 2-mercaptoethanol, followed by precipitation with 25% trichloracetic acid (TCA), dissolution in SDS-PAGE sample buffer, and boiling. Fractionation of cells was performed by differential centrifugation according to [38] with modifications. First, sedimented cells were suspended in 0.1 M Tris-SO_4_ buffer (pH 9.3) containing 0.5 M 2-mercaptoethanol and incubated for 10 min at 30 °C. After sedimentation, cells were rinsed with 1.2 M sorbitol in 20 mM potassium phosphate buffer (pH 7.4) and suspended in the same buffer containing 1 mg/ml Zymolyase 100T (Seikagaku) and digested for 90 min at 30 °C. Spheroplasts were sedimented and rinsed with 1.2 M sorbitol, suspended in 0.6 M sorbitol in 20 mM potassium phosphate buffer (pH 7.4) containing protease inhibitors cOmplete (Roche) and 1 mM PMSF, phosphatase inhibitors [25 mM 3-glycerophosphate, 5 mM Na_3_VO_4_, 10 mM NaF (all Sigma)] and deSUMOylation inhibitor 20 mM *N*-ethylmaleimide (Sigma). Subsequent procedures were carried out at 4 °C or on ice. Spheroplasts were disrupted in a Potter homogenizer. The nuclei-enriched fraction was sedimented by centrifugation at 3000×*g* for 5 min. Resulting supernatant was subsequently centrifuged at 13,000×*g* for 10 min producing a pellet enriched in mitochondria and peroxisomes and supernatant representing cytosol. Proteins from whole cells or nuclei—enriched fractions were extracted according to [39] with some modifications. Briefly, pellets were lysed in 1.85 M NaOH containing 7.4% 2-mercaptoethanol. The lysate was divided into two equal volumes and protein was precipitated by addition of an equal volume of 50% TCA. One precipitate was dissolved in a buffer containing 3 M urea and 1% SDS and used for determination of protein concentration using Lowry method [40], while the other was dissolved in an equal volume of Laemmli sample buffer. To adjust protein concentration samples were diluted appropriately with the same buffer. Proteins from the cytosolic fraction were precipitated with TCA added to a final concentration of 10% and prepared as above. Equal volumes of samples, i.e., equal amounts of total protein per lane (85 μg) were loaded on SDS-PAGE. To visualize GFP—tagged proteins samples were subjected to 12% SDS-PAGE followed by blotting onto Hybond-C extra membrane and probing with an anti-GFP antibody (Roche, Cat. No. 11,814,460,001) diluted 1:1000. To visualize histone H3 anti-Histone H3 (rabbit polyclonal) antibodies (Millipore, Cat. No. 2424672) diluted 1:5000 were used. Secondary anti-mouse (Invitrogen, Cat. No. WP20006) or anti-rabbit (Anti-Rabbit IgG (Fc), Promega, Cat. No. S373B) alkaline phosphatase-conjugated antibodies diluted 1:7500 were used. Western Blue Stabilized Substrate for Alkaline Phosphatase (Promega, Cat. No. S3841) was used to detect proteins. The membrane was scanned and band intensity was measured using Fiji ImageJ (1.50i) software [41].

### Co-immunoprecipitation

YPD-grown cells were subjected to nitrogen starvation in SD-N medium. A volume of yeast culture representing 100 OD_600_ units was collected and rinsed with homogenization buffer (50 mM Tris–HCl pH 7.5, 50 mM NaCl, 0.2% Triton X-100) and suspended in 1 ml of the same buffer supplemented with protease inhibitors [cOmplete (Roche) and 1 mM PMSF]. Cells were disrupted in homogenization tubes (Roche, #03358941001) in a MagNA Lyser instrument (Roche, #03358976001) for three cycles (6500 RPM, 50 s) separated by incubation on ice for 1 min. The homogenate was spun down (20,000×*g*, 10 min), anti-RFP (MBL, #M165-9) or anti-GFP (MBL, #D153-9)-coated magnetic beads were added to the supernatant and incubated at 4 °C for 2 h with gentle agitation. Beads were collected with a magnet and rinsed 3 times with homogenization buffer, 50 μl of Laemmli sample buffer was added and samples were boiled for 5 min. Next, the beads were removed and samples were analyzed by Western blotting as above. Primary antibodies used were: rabbit anti-RFP (Living colors DsRed polyclonal antibody, Clontech #632496) diluted 1:1000 or rabbit anti-GFP (Living colors A.V. Peptide antibody, Clontech #632377) diluted 1:400. The secondary antibody and signal detection were as above.

### Fluorescence microscopy

A Carl Zeiss AxioImager M2 (MicroImaging GmbH) fluorescence microscope with Filter Set 20 HE (Carl Zeiss, Cat. No. 489020-0000-000) was used. Images were captured using AxioCam MRc 5 camera (Carl Zeiss). DNA was stained by 1-h incubation of cells in fresh growth medium supplemented with 2.5 μg/ml of DAPI (4′,6-diamidino-2-phenylindole), at 30 °C. For quantitation of GFP signal distribution between nucleus (N) and cytoplasm (C) signal intensity ratio was determined for 25 GFP-expressing randomly picked cells per each time indicated in experiment, from at least 5 viewing fields. Average pixel intensity was calculated using Fiji ImageJ (1.50i) software [41] for 2-μm diameter circle representing region of either compartment. Background pixel intensity was subtracted and the resulting values were divided (N/C) to give the signal strength ratio, which is shown as average ± SD.

## Results

### Irr1 has a dual subcellular localization

**Fig. 1:**
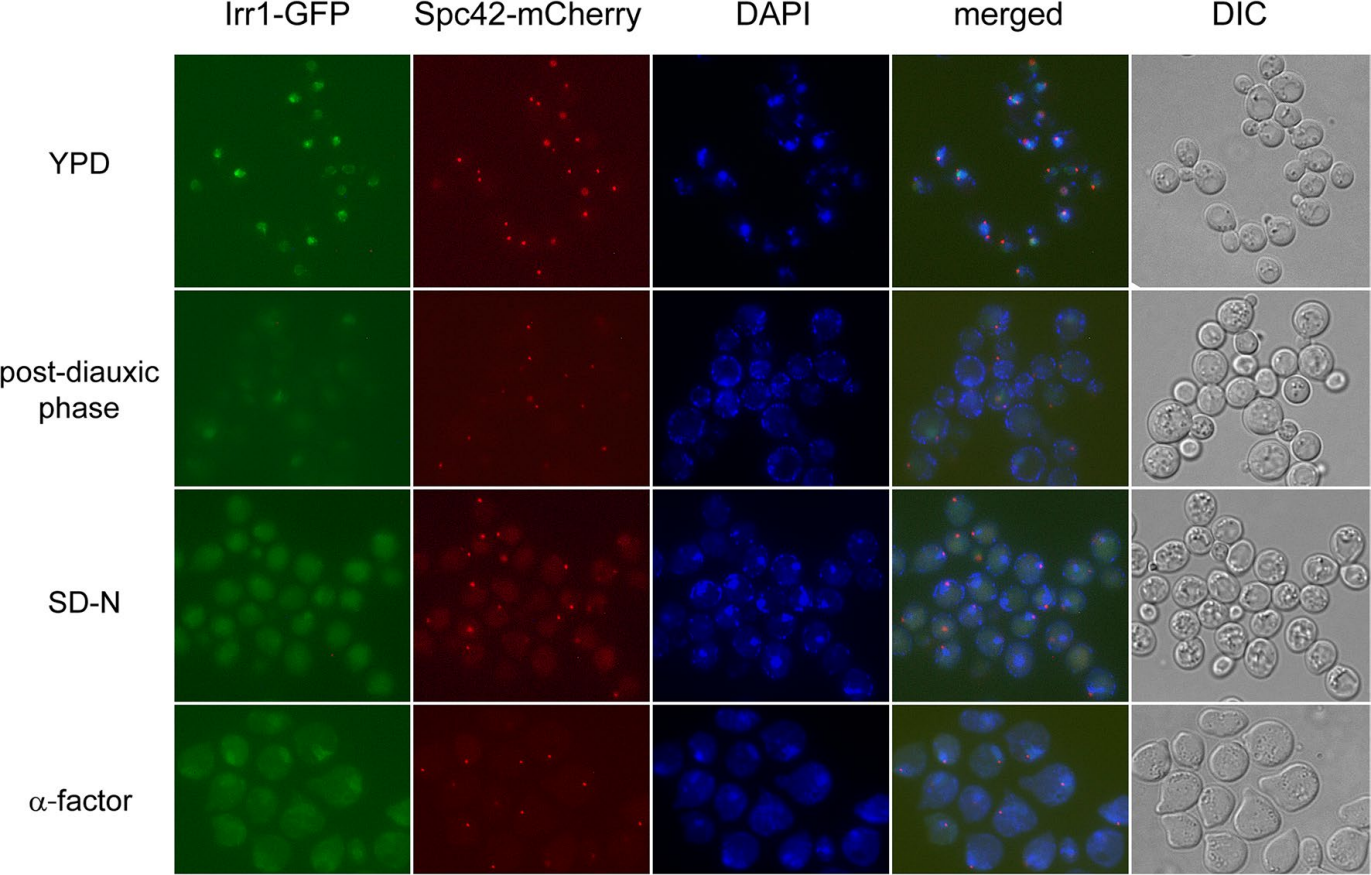
Irr1 protein can be present outside the nucleus. Growth arrest in G1 phase is accompanied by the appearance of Irr1 in the cytoplasm. *Rows*, *top* to *bottom*, show Irr1-GFP signal in cells in exponential phase of growth in YPD medium, in post-diauxic phase (ca. 24 h in YPD), following 22 h of nitrogen starvation in SD-N medium, and following α-factor treatment (6 μg/ml for 3 h). Spc42-mCherry—spindle pole body protein fused with mCherry, a nuclear marker, *DAPI* DNA stained with DAPI, *DIC* transmitted light

It is generally accepted that in dividing cells the Irr1 protein, being involved in chromosomal processes, is present in the nucleus. However, we noticed that in unsynchronized yeast cultures in some cells Irr1 was also present outside the nucleus. Studying various growth conditions we noticed that the change in Irr1 distribution appeared when the cells ceased exponential growth and was most pronounced in cells subjected to nitrogen starvation. This was in agreement with the Localization and Quantitation ATlas of the yeast proteomE (LoQAtE) data [32]. These observations indicated that a deficit of nutrients could be the cause of the appearance of Irr1 in the cytoplasm. Since it is known that starved yeast cells tend to arrest in the G1 phase [42], we checked whether the re-localization of Irr1 could also be achieved by blocking the cells in G1 phase using α-factor treatment instead of starvation. As shown in Fig. 1, that was indeed the case.

**Fig. 2:**
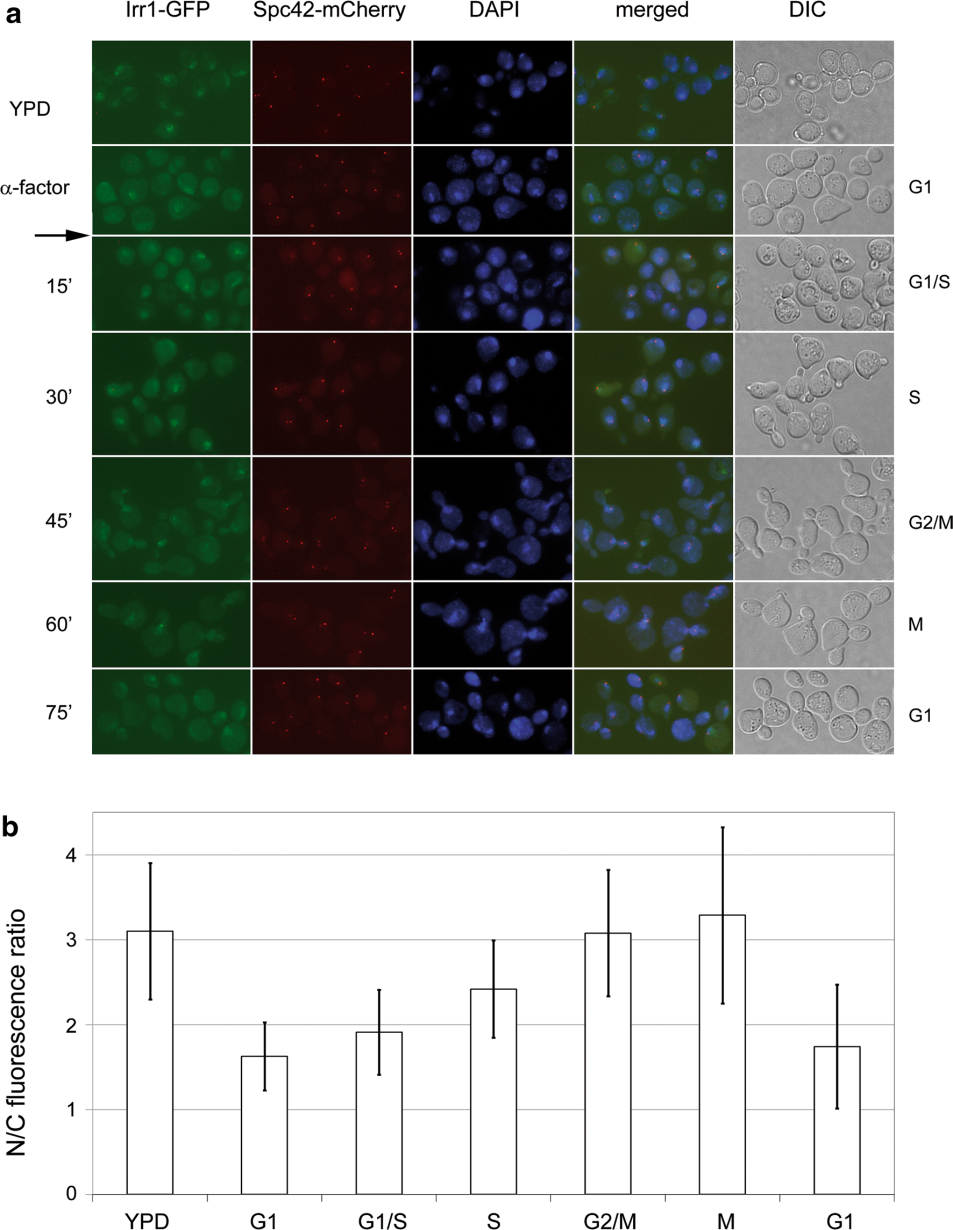
Irr1 appears in the cytoplasm in the G1 phase of the cell cycle. **a** Yeast culture was subjected to α-factor treatment as in Fig. 1, then allowed to resume the cell cycle by washing out the pheromone (*arrow*) and incubating the cells in YPD medium. At times indicated aliquots were withdrawn, fixed in formaldehyde and inspected for Irr1-GFP localization. Spc42-mCherry, DAPI and DIC as in Fig. 1. Cell-cycle phases inferred from cell and nuclear morphology are indicated on the right. **b** GFP fluorescence distribution between nucleus and cytoplasm. Signal intensity ratio was determined as described in “Methods” section and is shown as mean ± SD for 25 cells per experimental variant

While in exponentially growing cultures Irr1 was present almost exclusively in the nucleus, an increase of the proportion of cells in the G1 phase following depletion of nutrients (post-diauxic phase of growth), nitrogen starvation, or through cell-cycle stoppage by α-factor correlated with Irr1 appearance in the cytoplasm as well. To check whether the Irr1 relocation is associated with the cessation of growth or with the accumulation of cells in G1 we studied the nucleocytoplasmic distribution of Irr1-GFP throughout the cell cycle in an α-factor-synchronized culture (Fig. 2). Following the release of cells from α-factor action they resumed the cell cycle, which was accompanied by the disappearance of Irr1 from the cytoplasm. After mitosis and bud formation the cells entered the G1 phase and the cytoplasmic signal became well pronounced again. Thus, the appearance of Irr1 in the cytoplasm is a cyclic event related to the G1 phase and does not require growth arrest.

**Fig. 3:**
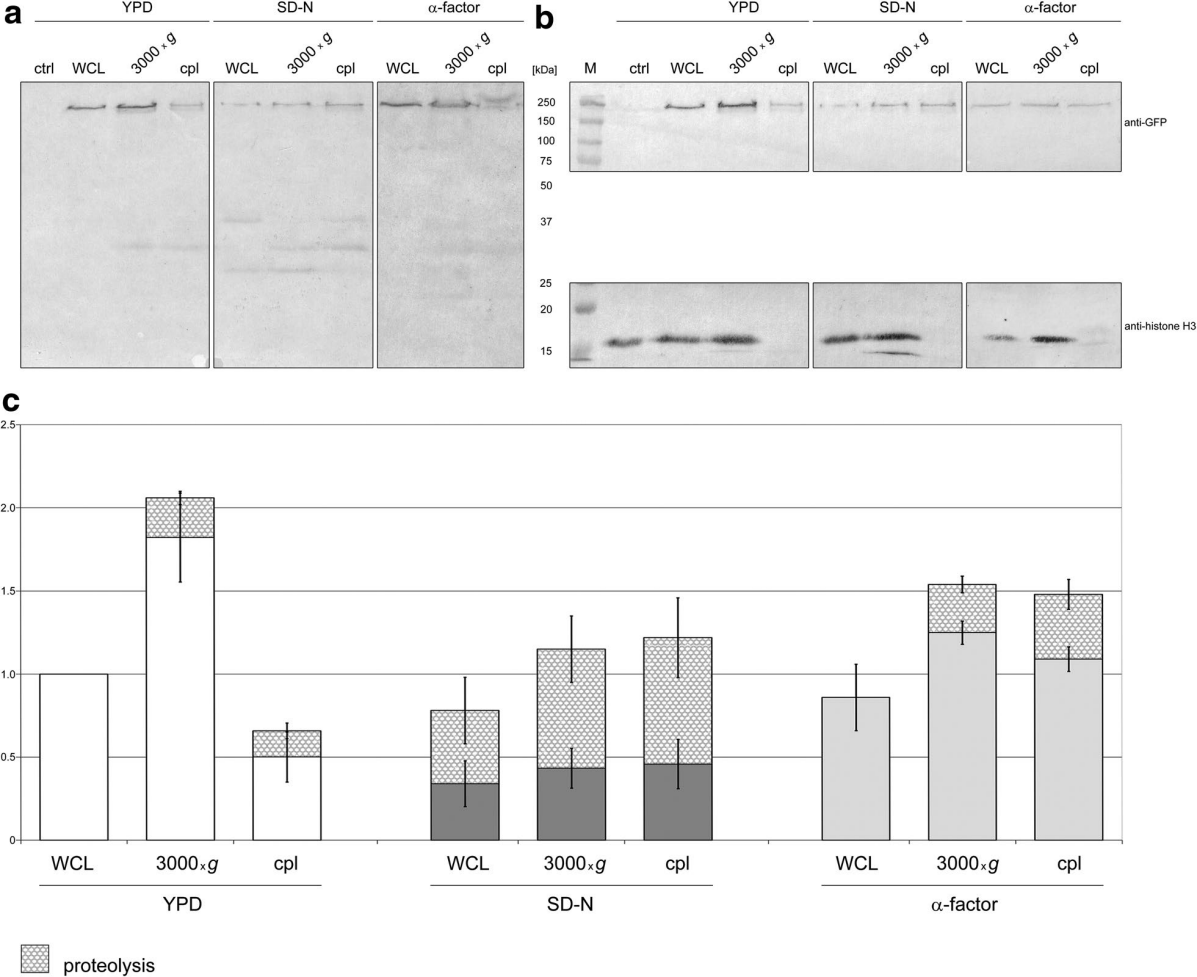
Nucleocytoplasmic distribution of Irr1 depends on growth conditions. Cells expressing Irr1-GFP were grown in YPD medium to exponential phase (YPD) or subjected to nitrogen starvation for 22 h (SD-N) or treated with α-factor for 3 h (α-factor) and whole-cell lysates (WCL) were prepared and fractionated into crude nuclear (3000×*g*) and cytoplasmic (cpl) fractions as detailed in “Methods” section. The unfractionated lysate and the fractions were separated by SDS-PAGE (85 μg of protein per lane), transferred to Hybond-C extra membrane, probed with anti-GFP antibodies (**a** and *top panel* in **b**) or anti-histone H3 antibodies (*bottom panel* in **b**), developed as detailed in “Methods” section and relative signal intensity was plotted taking WCL from YPD-grown cells as 1. All bands below that corresponding to intact Irr1-GFP are represented as “proteolysis” (**c**). All values are shown as mean ± SD of four independent experiments. The *two panels* in **b** represent the same membrane. Lane M contains molecular mass markers and ctrl is a negative control—whole cell lysate from cells expressing untagged Irr1. Western blots as in **a** were quantified using Fiji Image J software

To verify the subcellular localization of Irr1 indicated by microscopic observations we performed cell fractionation followed by Western blotting for Irr1-GFP for exponentially growing and growth-arrested cells (Fig. 3).

Unexpectedly we found that upon nitrogen starvation a substantial fraction of the chimaeric protein undergoes limited proteolysis, producing two bands of ca. 40 and 27 kDa, the latter likely representing GFP alone (M_w_ = 26,842 Da). These degradation products were present in the whole-cell extract obtained using a rapid and reliable procedure in the presence of a protease inhibitor cocktail and therefore are unlikely to have arisen during the extract preparation. No signs of such degradation were observed in cells grown in rich medium or those subjected to α-factor treatment. The overall level of Irr1-GFP in nitrogen-starved cells, as represented by the intact protein and the two degradation products, constituted ca. 80% of that in whole-cell extract from YPD-grown cells and ca. 90% in α-factor-treated cells. When the cells were fractionated into crude nuclear and cytoplasmic fractions, some degradation of Irr1-GFP was visible regardless of the cell growth conditions, we therefore integrated the GFP-derived signal from all the bands.

As per the microscopic analysis, also here the nucleocytoplasmic distribution of Irr1-GFP varied markedly between the actively growing and arrested cultures. In the rich medium the nuclear abundance (per mg total protein) of Irr1-GFP was ca. four times that in the cytoplasmic fraction, while in the SD-N medium or following α-factor action Irr1 showed roughly equal distribution between the two cell compartments. There was no contamination of the cytoplasamic fraction by nuclear material as evidenced by a lack of histone H3 (Fig. 3b, bottom panel). Since the Irr1-GFP degradation products could have leaked out from the nucleus, thereby artifactually increasing the apparent abundance of the GFP signal in the cytoplasm, we also calculated the distribution of intact, undegraded Irr1-GFP and obtained virtually identical results (see relevant bars representing undegraded protein in Fig. 3c).

Taken together, both the microscopic and the cell fractionation studies indicate that under certain conditions, such as nitrogen starvation or α-factor action, Irr1 changes its intracellular distribution from predominantly nuclear to roughly uniform nuclear-cytoplasmic. This redistribution coincides with the accumulation of cells in the G1 phase.

### Irr1 presence in cytoplasm does not result from Crm1—dependent export from nucleus

**Fig. 4:**
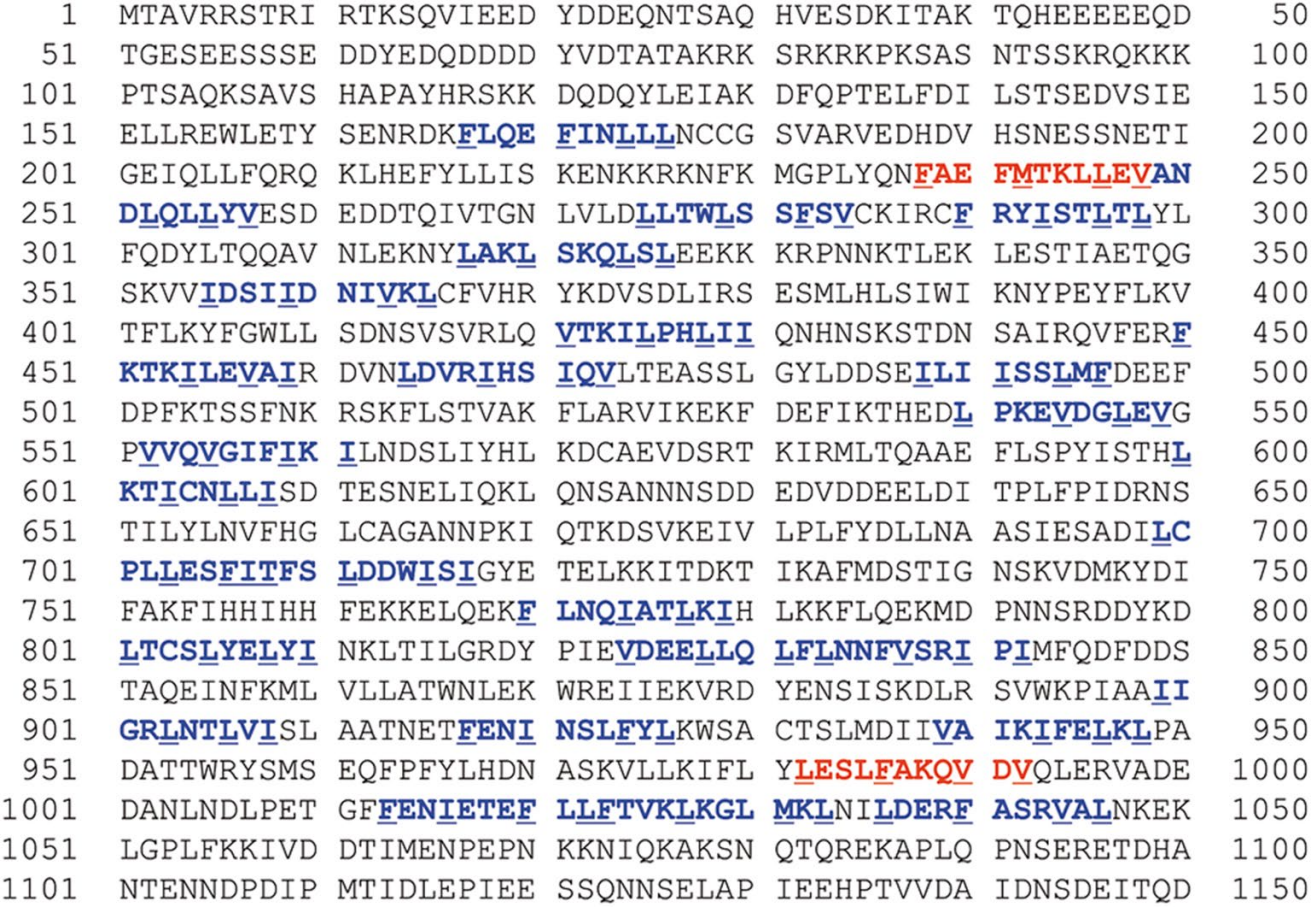
Irr1 protein contains multiple putative nuclear export signals (NES) recognized by Crm1 exportin. All NES signals are in *bold*, *red* signals identified by NetNES program, *blue* identified manually. *Underlined* hydrophobic amino acids crucial for interaction with Crm1

We showed before that SA2, a mammalian orthologue of Irr1, shuttles between the nucleus and the cytoplasm in a Crm1 exportin-dependent manner [28]. It was therefore likely that also Irr1 is exported from the nucleus upon cell cycle arrest. The presence of numerous putative sequence motifs for Crm1 in the amino acid sequence of Irr1 (Fig. 4) additionally supported the nuclear export concept. Among the 27 Crm1 consensus motifs found by manual inspection, two were also identified by the NetNES 1.1 prediction server [43].

**Fig. 5:**
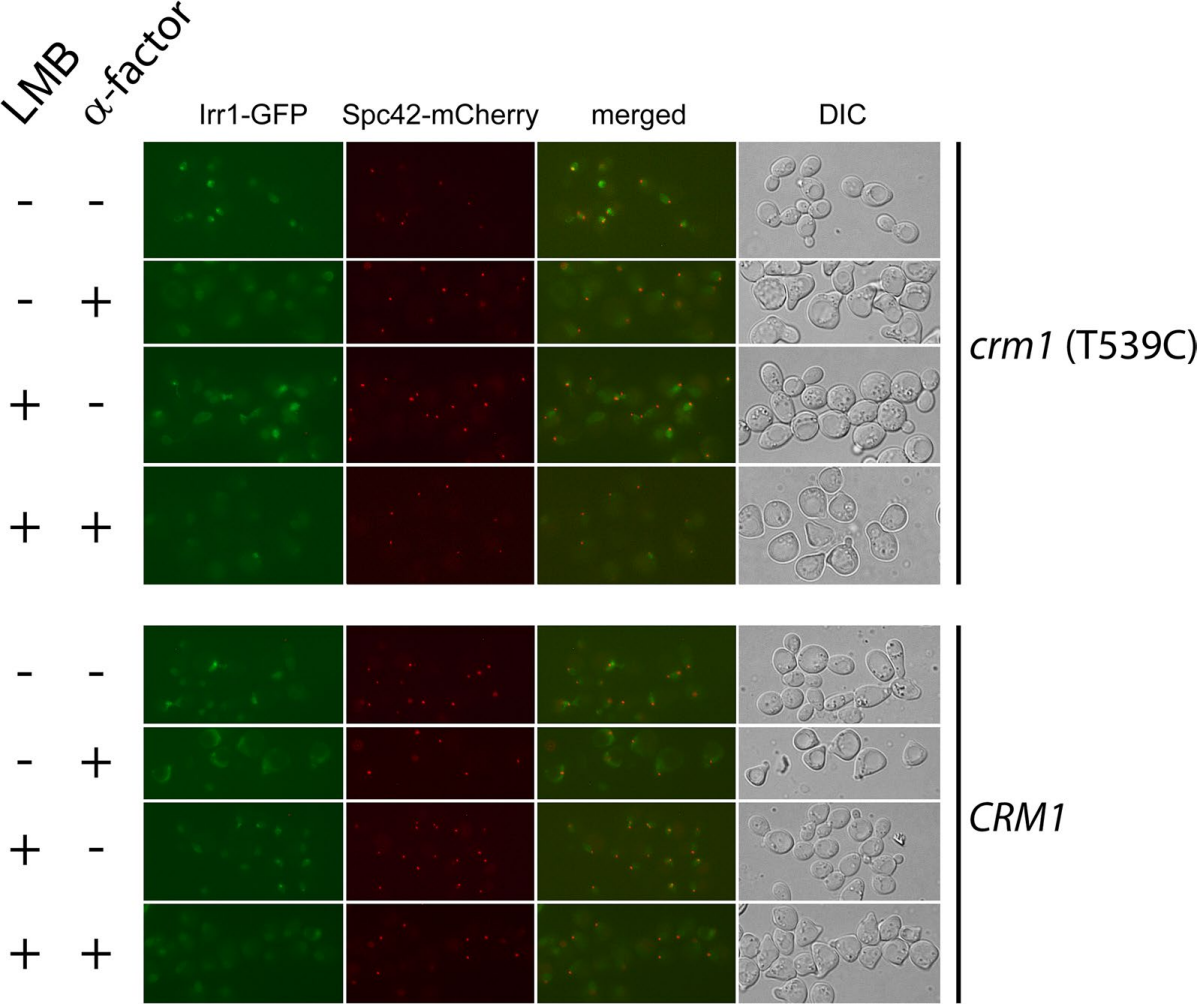
Cytoplasmic localization of Irr1 does not result from Crm1-dependent nuclear export. Subcellular localization of Irr1-GFP was analyzed after addition of LMB (Crm1 inhibitor) to 40 ng/ml to cells in logarithmic phase of growth or subjected to α-factor arrest. Strain *crm1*(T539C) bears LMB-sensitive version of Crm1p. *Spc42-mCherry* nuclear marker, *DIC* transmitted light

To check whether Irr1 is indeed exported from the nucleus we used the potent Crm1 inhibitor Leptomycin B (LMB) [44]. For this purpose we constructed a strain expressing Irr1-GFP and bearing the *crm1*-T539C mutation (MNY8-IRR1-GFP) which renders the exportin sensitive to LMB [34]. If the cytoplasmic localization of Irr1 involved Crm1—dependent export, then inhibition of Crm1 with LMB should lead to the retention of Irr1-GFP in the nucleus. Exponentially growing MNY8-IRR1-GFP cells were subjected to α-factor arrest in the presence or absence of LMB and inspected for the distribution of Irr1-GFP. In the both conditions the picture was the same, with a substantial cytoplasmic signal (Fig. 5). Thus, the cytoplasmic localization of Irr1-GFP in α-factor-arrested cells does not result from Crm1-dependent nuclear export.

**Fig. 6:**
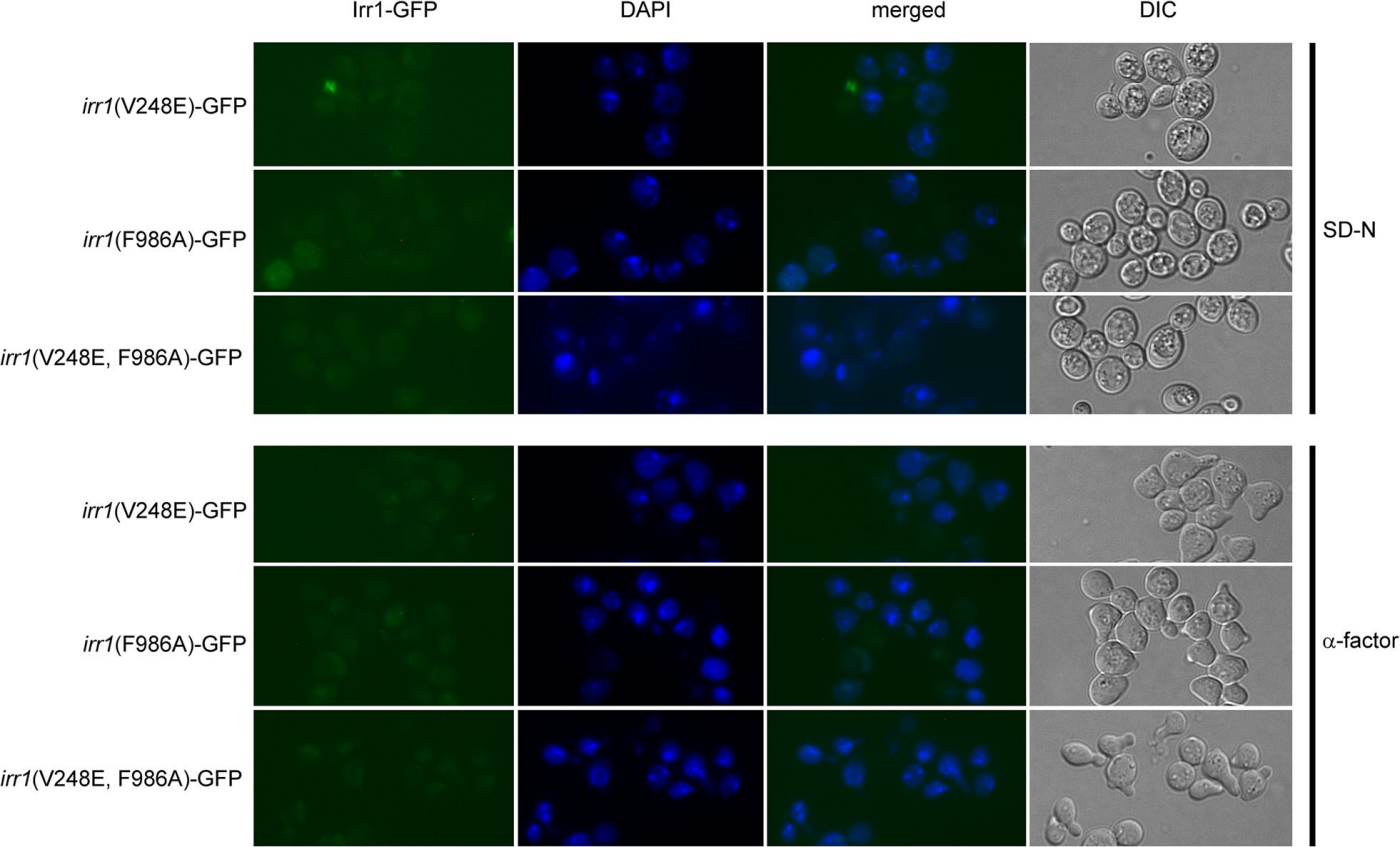
Distruction of two most likely NESs does not influence Irr1 cellular distribution upon nitrogen starvation or α-factor arrest. Cells expressing GFP-tagged Irr1 variants with one or two potential NES motifs disrupted, as indicated on the *left*, were grown in YPD medium and subjected to nitrogen starvation for 22 h (SD-N) or treated with α-factor for 3 h (α-factor). DAPI-DNA stained with DAPI, DIC—transmitted light. Consensus sequence for Crm1-dependent nuclear export is WX2–3WX2–3WXW, where *W* represents L, I, V, F or M and X—any amino acid. NES signals disrupted by site-directed mutagenesis. *irr1*(V248E) carries inactivated NES between positions 238–248, *irr1*(F986A)—between positions 982–992, *irr1*(V248E, F986A) has both signals inactivated

The above approach could not be used to study nuclear export of Irr1 upon nitrogen starvation because it would require LMB addition at the beginning of the 22-h starvation, causing cell death. We therefore used a different strategy and destroyed the two most likely NESs (in positions 238–248 and 982–992) identified in Irr1 by NetNES by introducing the V248E and/or F986A substitutions. All three mutated *IRR1*-*GFP* variants (two single and the double mutant) were functional, as the respective *irr1Δ* yeast strains bearing plasmids with mutated genes were viable. Upon nitrogen starvation or following α-factor action the manipulated proteins were distributed in the cell in exactly the same manner as was wild type Irr1-GFP (Fig. 6).

Thus, the presence of Irr1-GFP outside the nucleus under nitrogen starvation does not result from Crm1-dependent export. In summary, the presence of a large proportion of Irr1in the cytoplasm under certain conditions is likely due to its reduced nuclear import and/or enhanced cytoplasmic retention rather than to active export from the nucleus, although a Crm1-independent export pathway cannot be excluded.

### Irr1 interacts with cytoplasmic protein Imi1

We assumed that the cytoplasmic pool of Irr1 could play a role in non-cohesion-related processes. To identify possible cytoplasmic partners of Irr1 we performed a two-hybrid screen. Previous screens carried out in our laboratory employed full-length Irr1 protein or its N-terminal fragment as baits [45]. Both those baits contained the evolutionarily conserved STAG domain required for interaction with the well-characterized component of the cohesion complex, the Mcd1 protein [46]. We reasoned that by using an Irr1 fragment devoid of this domain as bait one could unmask possible interactions with proteins unrelated to sister chromatid cohesion thus revealing Irr1 function(s) outside the nucleus.

To this end we used a C-terminal part of Irr1 starting from I468 and constituting 59% of the protein. Two interacting proteins were identified, Mrps5, a mitochondrial ribosomal protein [47], and the cytoplasmic protein Imi1 involved in glutathione homeostasis and mitochondrial integrity [30] (Fig. 8a). The interaction with Imi1 indicated that Irr1 could indeed play a role in the cytoplasm, particularly under growth-disturbing conditions.

**Fig. 7:**
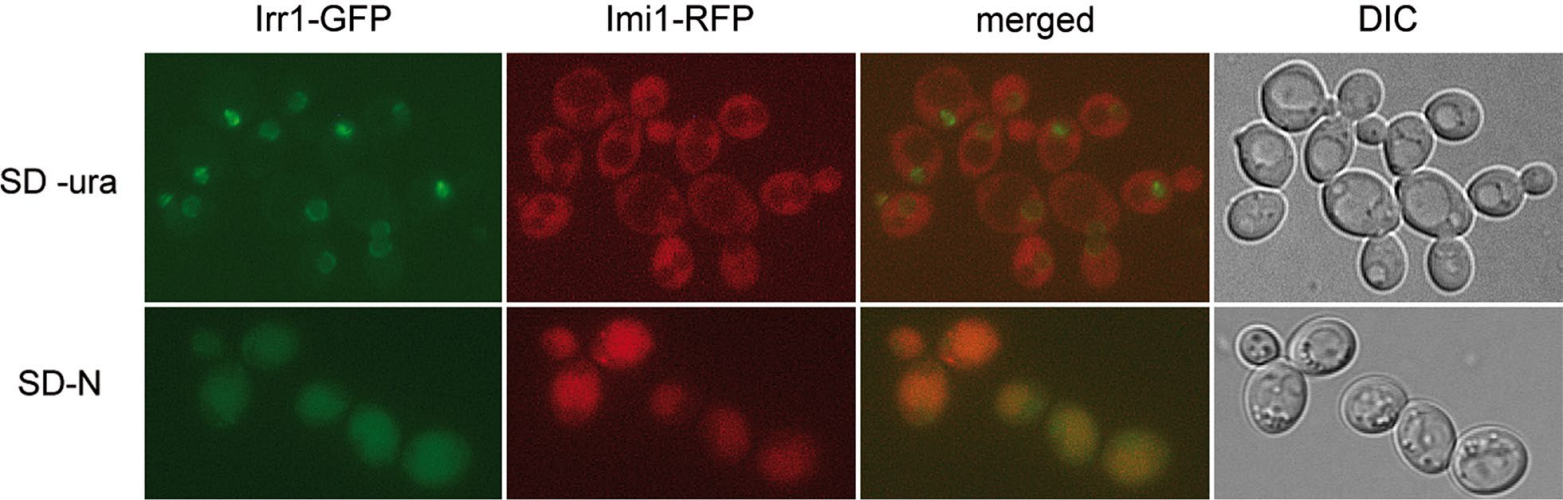
Imi1-RFP and Irr1-GFP partially co-localize upon nitrogen starvation. *Upper panel* Irr1-GFP/Imi1-RFP cells grown in SD-ura medium to exponential phase. *Lower panel* cells of the same strain subjected to nitrogen starvation for 22 h

To verify co-localization of these two proteins, we constructed a strain bearing fluorescently labelled full-length Irr1-GFP and Imi1-RFP and studied it under various growth conditions (Fig. 7). As expected from the earlier observations, in the exponential phase of growth Imi1 and Irr1 proteins did not co-localize, but when the cells were transferred to the nitrogen starvation medium and the distribution of Irr1 changed, the two proteins co-localized partially in the cytoplasm.

**Fig. 8:**
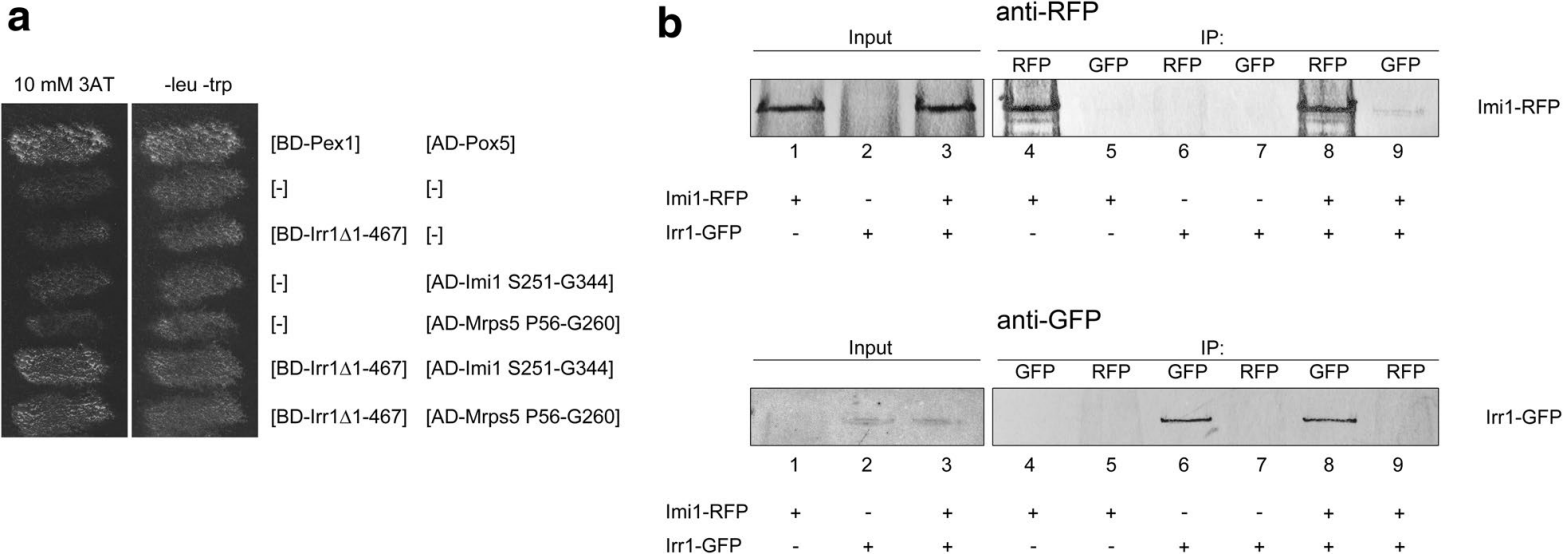
Irr1 physically interacts with Imi1. **a** Interaction of C-terminal part of Irr1 with Imi1 by two-hybrid assay. PJ69-4α strain was co-transformed with plasmids encoding bait and prey proteins as indicated and tested for growth on SD medium lacking histidine in the presence of 10 mM 3-amino-1,2,4-triazole. Plasmids encoding BD-Gal4and AD-Gal4 were used as negative controls [−], whereas a strain bearing BD-Pex1 and AD-Pox5 served as a strong positive control. The interacting fragment corresponds to amino acids from S251 to G344 of Imi1. Interaction with the second identified protein, Mrps5 (fragment comprising amino acids P56-G260), is also shown. **b** Irr1-GFP and Imi1-RFP co-precipitate. SD-ura-grown cells were subjected to nitrogen starvation for 22 h. Cells were homogenized and proteins were precipitated using magnetic beads coated with anti-GFP or anti-RFP antibodies, as indicated (IP). Immunoprecipitates were separated by SDS-PAGE, transferred to Hybond-C extra membrane and probed with anti-RFP (*upper panel*) or anti-GFP (*lower panel*) antibodies. Strains used were: negative controls GFP/Imi1-RFP (*lanes 1*, *4* and *5*) and Irr1-GFP/RFP (*lanes 2*, *6* and *7*), and the study strain Irr1-GFP/Imi1-RFP (*lanes 3*, *8* and *9*)

We further confirmed the Irr1–Imi1 interaction for the full-length intact proteins by co-immunoprecipitation using the same strain and nitrogen-starvation conditions (Fig. 8b).

Due to the fact that upon expression from original promoter Imi1 is undetectable [30] and Irr1 is also not an abundant protein, the level of coprecipitation is rather low. However, it indicates that it is likely that, when in the cytoplasm, Irr1 could form a complex with the metabolic regulator Imi1.

## Discussion

We report here a cytoplasmic localization of the protein Irr1 known so far as a regulator of sister chromatid cohesion acting in the nucleus only. The cytoplasmic localization is observed in growth-arrested yeast cells following nutrient depletion, nitrogen starvation or α-factor effect. Starvation for nitrogen, as well as for carbon, phosphate or sulfur triggers exit from the cell cycle in G1 phase and entry into a quiescent state (G0) [48, 49]. Also α-factor arrests the cells in G1 phase in preparation for mating. In fact, we found that a roughly uniform nucleo-cytoplasmic distribution of Irr1 is a feature of the G1 phase even in the absence of growth arrest. Notably, in G1-synchronized rat cells (NRK line) cohesins are either not associated with chromatin or in a dynamic state, albeit their non-nuclear localization has not been reported [7].

Similarly to Irr1, also other proteins predominantly involved in nuclear processes, among them cohesins Smc3 and Mcd1, can have additional functions outside the nucleus. It has been shown that Smc3 can function as bamacan, a proteoglycan assembled into basement membranes in mammals [50]. The second cohesin, Mcd1, is specifically proteolyzed by caspases in cells undergoing apoptosis in response to diverse stimuli and its carboxy-terminal product amplifies the cell death signal in mitochondria [11, 12].

The G1 phase is a pivotal moment in the cell cycle where decision whether to begin the metabolically costly S phase, become quiescent to save nutrients or prepare for mating is made. At that time Irr1 likely interacts in the cytoplasm with a newly-identified regulator of glutathione metabolism and mitochondrial functioning, Imi1 [30]. Glutathione is involved in the physiological response to various stresses, including nitrogen starvation. Upon starvation, glutathione is shifted to the central vacuole and then degraded releasing its constituent amino acids Glu, Cys and Gly into the cytosol [51, 52]. On the other hand, some reports have indicated that maintenance of glutathione concentration is a general priority for cells under conditions of a dearth of glutathione precursors [53, 54].

Upon nitrogen starvation of yeast cells cellular respiration strongly decreases and mitophagy is initiated, excess mitochondria are degraded, and production of reactive oxygen species (ROS) in mitochondria is suppressed [55]. Despite that knowledge, the perturbations in the ROS metabolism that could trigger cell cycle checkpoints and dictate cell fates (e.g., G1 delay) remain largely unexplored [56]. It is an interesting possibility that through interaction with Imi1 Irr1 affects the glutathione homeostasis and thereby the overall cell metabolism thus assisting the choice of the cell fate depending on external conditions. Remarkably, when nutrients are limiting Irr1 is heavily proteolyzed which decreases its cytoplasmic pool relative to that in cells in growth-promoting conditions.

## Conclusions

In addition to its nuclear function as a regulator of the sister chromatid cohesion complex the Irr1/Scc3 protein abounds in the cytoplasm in the G1 phase of the cell cycle in both actively dividing and growth-arrested cells. There Irr1 likely interacts with Imi1, a regulator of glutathione homeostasis and mitochondrial functioning. On nitrogen limitation Irr1 undergoes partial proteolysis. These features suggest a role for Irr1 in modulating the cell metabolism during a crucial step of the cell cycle.

## Abbreviations

DAPI: 4′,6-diamidino-2-phenylindole dihydrochloride
GFP: green fluorescent protein
LMB: leptomycin B
NLS: nuclear localization signal
NES: nuclear export signal
